# In Vitro Antimicrobial Potential of Different Platelet Concentrates Against Eight Clinically Relevant Oral Pathobionts

**DOI:** 10.3390/antibiotics15020173

**Published:** 2026-02-05

**Authors:** Ellen E. Jansen, Zahra Hejazi, Andreas Braun, Patrick Jansen, Georg Conrads

**Affiliations:** 1 Department of Operative Dentistry, Periodontology and Preventive Dentistry, Rheinisch-Westfälische Technische Hochschule (RWTH) University Hospital, 52074 Aachen, Germany; 2 Division of Oral Microbiology and Immunology, Department of Operative Dentistry, Periodontology and Preventive Dentistry, Rheinisch-Westfälische Technische Hochschule (RWTH) University Hospital, 52074 Aachen, Germany

**Keywords:** platelet concentrates, platelet-rich plasma, platelet-rich fibrin, injectable PRF, antimicrobial activity, oral microorganisms, agar diffusion testing

## Abstract

**Background/Objectives:** Oral infections are caused by a wide spectrum of bacterial and fungal species and remain clinically challenging, particularly against the background of increasing antimicrobial resistance and efforts to reduce antibiotic use in dentistry. Platelet concentrates are widely applied in periodontal and oral surgery due to their regenerative and immunomodulatory properties, and accumulating evidence suggests additional antimicrobial effects. This study evaluated the antimicrobial activity of platelet-rich plasma (PRP), platelet-rich fibrin (PRF), and injectable PRF (i-PRF) against clinically relevant oral microorganisms. **Methods:** PRP, PRF, and i-PRF were prepared from venous blood of five healthy donors and evaluated using diffusion-dependent, qualitative-semiquantitative agar diffusion assays against *Aggregatibacter actinomycetemcomitans*, *Porphyromonas gingivalis*, *Prevotella intermedia*, *Staphylococcus aureus*, *Streptococcus mutans*, *Streptococcus mitis*, *Enterococcus faecalis*, and *Candida albicans*, with inhibition zones assessed after species-specific incubation times. Chlorhexidine (2%) and amoxicillin served as positive controls and NaCl (0.9%) as negative control. Inhibition zones were digitally quantified and analyzed using non-parametric statistics (Kruskal–Wallis, Friedmann) due to skewed distributions and frequent zero values. **Results:** All platelet concentrates demonstrated microorganism-dependent inhibition zones in vitro. Overall, i-PRF demonstrated the strongest inhibitory effect across all pathogens (*p* < 0.001). Significant differences were detected for *E. faecalis* and *C. albicans*, where i-PRF produced markedly larger inhibition zones compared to PRP and PRF. Descriptively, anaerobic periodontal pathogens and *S. aureus* tended to be more susceptible, while streptococci and *C. albicans* demonstrated lower inhibition. **Conclusions:** These findings support a potential adjunctive antimicrobial role of platelet-derived preparations in dental infection management but should be interpreted with caution, as agar diffusion results do not necessarily reflect clinical performance.

## 1. Introduction

Infections in the oral area continue to pose a considerable challenge despite modern therapeutic options. In addition to classic bacterial pathobionts such as *Streptococcus mutans* (*S. mutans*) or *Porphyromonas gingivalis* (*P. gingivalis*), opportunistic pathogenic fungi such as *Candida albicans* (*C. albicans*) are also becoming increasingly important. Oral infections are not only locally problematic, but the periodontium also serves as a critical epithelial and immunological barrier that limits dissemination of oral microorganisms into the systemic circulation [1]. When this barrier is compromised, as in chronic periodontal disease, oral pathogens can have broader impacts on overall health, contributing to conditions such as cardiovascular disease or diabetes mellitus [2]. In view of the increasing antibiotic resistance, which is classified by the WHO as one of the greatest global health threats [3], there is growing interest in alternative strategies for oral infection control.

Platelet concentrates have traditionally been used intravenously in human medicine for the treatment of thrombocytopenias and thrombocytopathies [4]. In recent years, however, it has been shown that the local application of platelet-rich concentrates and especially platelet-rich fibrin (PRF) can promote wound healing and tissue regeneration [5]. Autologous platelet preparations have therefore increasingly found their way into dental practice. They are used, among other adjunctive therapeutic approaches, in periodontal and oral surgery, e.g., to support bone healing after extractions, sinus lift operations, apicoectomies, or in surgical periodontal therapy [6,7]. Beyond their regenerative capacity, platelet concentrates also exhibit immunomodulatory biological activity that depends on preparation protocols, cellular composition, and fibrin architecture [8].

Platelets make up about 1.7% of blood and are best known for their role in haemostasis and wound healing, but they also represent active components of the innate immune system [9]. They express a broad range of pathogen recognition receptors, including Toll-like receptors (TLR2, TLR4, and TLR9), and can directly interact with microorganisms. Upon activation, platelets undergo morphological changes and release numerous bioactive mediators, such as cytokines, growth factors, antimicrobial peptides (e.g., CXCL4, CXCL7, and β-defensins), and reactive oxygen species, which contribute to direct antimicrobial effects and the recruitment of other immune cells [10,11]. In addition, platelets can stimulate neutrophil extracellular trap (NET) formation and interact with immunoglobulins and complement factors, thereby linking innate and adaptive immune responses [10].

In addition to their regenerative properties, the potential antimicrobial effects of platelet concentrates are also coming into focus. Initial in vitro studies indicate that platelet-derived preparations can inhibit bacterial and fungal growth. For example, Aggour et al. (2017) [12] described inhibitory effects of PRP on *Aggregatibacter actinomycetemcomitans* (*A. actinomycetemcomitans*), *P. gingivalis*, and *C. albicans*. Drago et al. (2013) [13] reported growth inhibition of *Enterococcus faecalis* (*E. faecalis*), *C. albicans*, *Streptococcus agalactiae*, and *Streptococcus oralis* by pure PRP. In addition, PRP was shown to significantly reduce the growth of periodontal pathogens such as *P. gingivalis* and *A. actinomycetemcomitans* in the time-kill assay [14]. A comparison of PRP and PRF also showed that only PRP showed antimicrobial activity [15]. Recent systematic reviews and narrative evaluations highlight antimicrobial activity of PRF variants, including i-PRF, across a range of in vitro models [16]. However, the reported studies used heterogeneous preparation protocols, investigated different microbial spectra, and applied various antimicrobial models (e.g., agar diffusion, time-kill, or biofilm assays), which hampers direct comparison and underscores the need for standardized, systematic evaluation. More recently, i-PRF, a leukocyte-rich liquid formulation produced at low centrifugal forces, has likewise demonstrated in vitro antimicrobial and antibiofilm effects, at least against oral pathogenic staphylococcal species [17].

Despite these first promising results, systematic analyses of the efficacy of platelet concentrates against oral pathogens that are particularly relevant for dentistry are still lacking. Because oral infections are usually polymicrobial and involve microorganisms with different susceptibilities, evaluation across multiple species is essential to obtain a clinically meaningful assessment of antimicrobial potential. Moreover, existing evidence is mainly based on in vitro studies with limited standardization, and direct comparisons between PRP, PRF, and i-PRF as well as systematic testing across the same panel of clinically relevant microorganisms within a single experimental framework are scarce. Consequently, the relative antimicrobial performance of different platelet preparations against clinically relevant oral microorganisms remains insufficiently defined.

The aim of the present study was to systematically evaluate and compare the antimicrobial potential of PRP, PRF, and i-PRF using agar diffusion testing against selected oral microorganisms. Specifically, we aimed (i) to assess microorganism-specific susceptibility patterns, (ii) to compare preparation-dependent differences in growth inhibition, and (iii) to determine the overall inhibitory profile of each platelet concentrate.

## 2. Results

A total of 120 inhibition zone measurements (averaged diameters from technical triplicates) were obtained. Due to the right-skewed distribution of the data with a substantial proportion of zero values, results are reported as medians and interquartile ranges (IQR), and non-parametric statistical tests were applied. For aggregated analyses, inhibition zone diameters were calculated per donor and preparation as the median across all tested microorganisms. For the negative control (0.9% NaCl), no inhibition zones were observed for any microorganism. Chlorhexidine (2%) consistently produced large zones of complete growth inhibition for all tested microorganisms, and for amoxicillin extensive growth inhibition up to almost complete clearing of the agar surface was observed on some plates. Due to these uniformly strong or extensive effects, reliable quantitative measurement was not possible and therefore control data were not included in the statistical analysis.

### 2.1. Preparation-Dependent Differences

**Figure 1:**
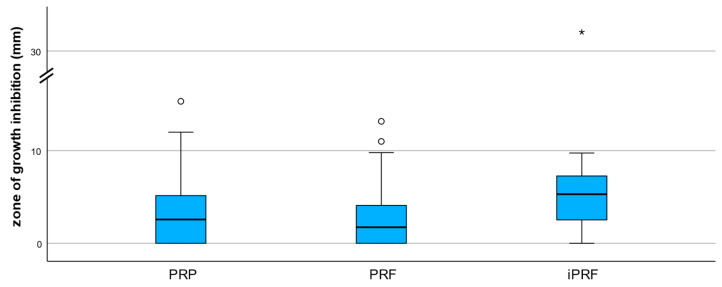
Boxplot of aggregated zones of growth inhibition (median across all microorganisms tested per donor and preparation). Mild outliers (circles) and extreme outliers (asterisks) are displayed to illustrate data variability. The y-axis includes a scale break to improve readability due to extreme values.

The inhibitory effect differed descriptively between the examined preparations. i-PRF had the highest median inhibition zones (median = 5.30 mm; IQR = 4.76 mm), followed by PRP (median = 2.57 mm; IQR = 5.24 mm) and PRF (median = 1.73 mm; IQR = 4.12 mm). All preparations showed a right-skewed distribution of the inhibitory zones with sometimes pronounced outliers, especially in i-PRF, which indicates a high variability of the inhibitory effect (shown in Figure 1).

### 2.2. Microorganism-Specific Susceptibility

**Figure 2:**
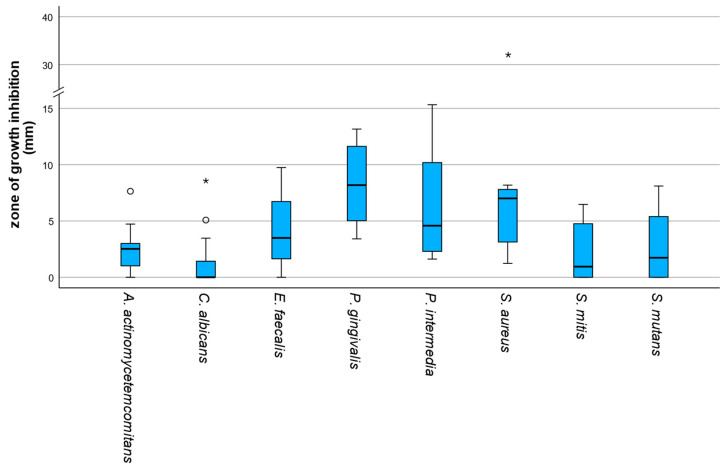
Boxplots of zones of growth inhibition (mm) by microorganism across all three preparations. Mild outliers (circles) and extreme outliers (asterisks) are displayed to illustrate data variability. The y-axis includes a scale break to improve readability due to extreme values.

There were significant differences in sensitivity to the preparations between the microorganisms examined (Friedman test, χ^2^(7) = 29.98, *p* < 0.001). High median growth inhibition zones were observed for *P. gingivalis* (median = 8.18 mm; IQR = 6.87 mm) and *Staphylococcus aureus* (*S. aureus*) (median = 7.01 mm; IQR = 5.36 mm). Intermediate median inhibitory zones were observed for *E. faecalis* (median = 3.50 mm; IQR = 5.32 mm), *Prevotella intermedia* (*P. intermedia*) (median = 4.58 mm; IQR = 8.54 mm) and *A. actinomycetemcomitans* (median = 2.52 mm; IQR = 2.50 mm). In contrast, *C. albicans* (median = 0.00 mm; IQR = 2.85 mm), *Streptococcus mitis* (*S. mitis*) (median = 0.94 mm; IQR = 4.93 mm) and *S. mutans* (median = 1.73 mm; IQR = 5.84 mm) exhibited low median inhibition zones, with frequent zero values. Data is shown in Figure 2.

### 2.3. Individual Susceptibility: Preparation vs. Microorganism

**Figure 3:**
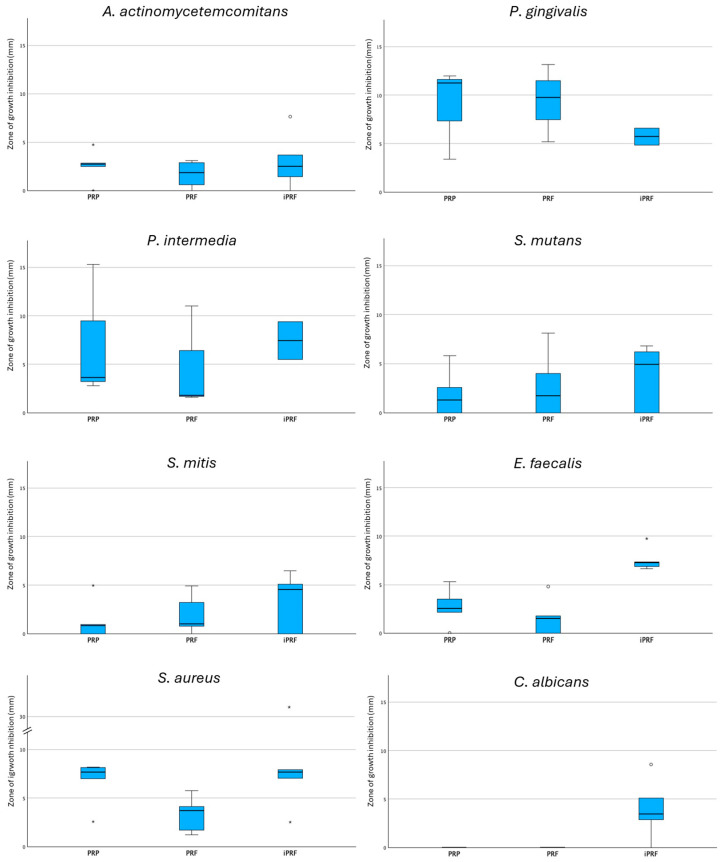
Boxplots showing the distribution of growth inhibition zone diameters (mm) for the eight investigated microorganisms following application of platelet-rich plasma (PRP), platelet-rich fibrin (PRF), and injectable platelet-rich fibrin (i-PRF). Boxes represent the interquartile range (IQR), the horizontal line indicates the median, and whiskers denote the minimum and maximum values. Mild outliers (circles) and extreme outliers (asterisks) are displayed to illustrate data variability. The y-axis of *S. aureus* includes a scale break to improve readability due to extreme values.

For an exploratory overview, the zone-diameters of growth inhibition were additionally combined according to preparation and microorganism. A box plot with all microorganisms and preparations (Figure 3) made it clear that the inhibitory effect of the preparations differs depending on the respective microorganism. i-PRF tended to exhibit higher median growth inhibition diameters for several microorganisms, whereas PRF frequently showed lower values, with considerable overlap between groups and substantial within-group variability (Figure 3). At the same time, a sometimes-considerable dispersion of the measured values within individual microorganism-preparation combinations became visible. Representative agar plate images illustrating typical inhibition patterns are provided in Supplementary Figure S1.

To investigate whether the inhibitory zones between the preparations within a microorganism differ, separate Kruskal–Wallis tests were carried out for each microorganism (Table 1). For most of the microorganisms examined, there were no statistically significant differences between PRP, PRF and i-PRF (*P. gingivalis*: *p* = 0.574; *P. intermedia*: *p* = 0.566; *A. actinomycetemcomitans*: *p* = 0.826; *S. mitis*: *p* = 0.688; *S. mutans*: *p* = 0.766). For *S. aureus*, there was a trend of differences between the preparations, but this did not reach the significance level (*p* = 0.067). Pairwise comparisons suggested higher inhibition for i-PRF versus PRF (*p* = 0.0472, r = 0.63); however, this difference did not remain significant after Bonferroni correction (*p*_adj ≈ 0.142). For *P. gingivalis* and *P. intermedia*, some plates were not evaluable due to insufficient growth under anaerobic conditions. Consequently, the number of evaluable donor-level observations was reduced for these species (PRP and PRF: *n* = 3; i-PRF: *n* = 2).

In contrast, there were significant differences between the preparations for *C. albicans* (*p* = 0.007) and *E. faecalis* (*p* = 0.007). For *C. albicans*, post hoc tests were omitted due to a lack of variance in the PRP and PRF groups (median = 0 mm). Nevertheless, pairwise comparisons indicated higher inhibition for i-PRF compared with PRP and PRF (each *p* = 0.0186, r = 0.74), although these comparisons were borderline after Bonferroni correction (*p*_adj ≈ 0.056). For *E. faecalis*, post hoc Mann–Whitney U tests with Bonferroni correction were performed, which showed that i-PRF had significantly higher inhibition zones than PRP (*p* = 0.008) and PRF (*p* = 0.008), while there was no significant difference between PRP and PRF (*p* = 0.31). These comparisons remained significant after Bonferroni correction (*p*_adj ≈ 0.027), with large effect sizes (r = 0.83 for both i-PRF vs. PRP and i-PRF vs. PRF). Regarding the question of whether inhibitory effects differ within each preparation across microorganisms, separate Friedman tests were performed for PRP, PRF and i-PRF. For none of the preparations there were significant differences in the inhibitory zones between the microorganisms (PRP: χ^2^(7) = 13.17, *p* = 0.068; PRF: χ^2^(7) = 13.12, *p* = 0.069; i-PRF: χ^2^(7) = 11.61, *p* = 0.114).

### 2.4. Overall Effectiveness of the Platelet Preparations

When evaluating the overall effectiveness of the preparations, the median inhibition zone-diameters across all microorganisms examined were calculated for each test subject and each preparation. The comparison of these aggregated inhibition zones using the Kruskal–Wallis test revealed a highly significant difference between the preparations (H(2) = 80.75, *p* < 0.001). i-PRF showed the highest mean ranks and thus had the strongest overall efficacy across all microorganisms, followed by PRP and PRF. Finally, to determine whether the microorganisms differed in their general susceptibility to platelet-preparations, a Friedman test was performed. This revealed a highly significant difference between the microorganisms (χ^2^(7) = 29.98, *p* < 0.001). The analysis of the mean ranks showed that *P. intermedia* and *P. gingivalis* had the highest inhibition zones, while *C. albicans* showed the lowest inhibition. The remaining microorganisms took intermediate positions.

## 3. Discussion

The present investigation demonstrates that platelet-derived concentrates exert a detectable antimicrobial effect against a spectrum of clinically relevant oral pathobionts. Importantly, this effect was not uniform but varied according to both the type of platelet preparation and the microorganism examined. These results indicate that the antimicrobial potential of platelet concentrates represents a differentiated, preparation-specific phenomenon rather than a nonspecific or generalized antimicrobial action.

### 3.1. Preparation-Dependent Antimicrobial Effects

Among the preparations evaluated, i-PRF exhibited the most pronounced overall inhibitory activity. Across all tested microorganisms, i-PRF not only resulted in a greater extent of growth inhibition but also showed a more consistent inhibitory pattern, whereas PRP and PRF displayed smaller and more variable effects. In contrast, the differences observed between PRP and PRF were comparatively limited, suggesting that antimicrobial efficacy is not solely determined by the presence of a fibrin matrix but rather by the combined effects of cellular composition and release kinetics of bioactive mediators [5,13].

When individual microorganisms were analyzed separately, statistically significant differences between platelet preparations were detected only for selected species. In particular, *E. faecalis* showed significantly greater susceptibility to i-PRF compared with PRP and PRF, whereas for most other microorganisms no significant preparation-related differences were identified. This observation suggests that certain bacterial species may respond more strongly to the specific cellular and molecular composition characteristic of i-PRF.

### 3.2. Species-Specific Susceptibility Patterns

Irrespective of the preparation used, clear differences in intrinsic susceptibility were observed among the investigated microorganisms. Obligate anaerobic periodontal pathogens such as *P. gingivalis* and *P. intermedia*, as well as the facultative anaerobic *S. aureus*, generally exhibited larger inhibition zones in the present dataset. In contrast, *C. albicans* and *S. mitis* consistently demonstrated low median inhibition across all preparations. These variations may reflect species-specific differences in cell wall composition, metabolic activity, and inherent resistance mechanisms, including the complex fungal cell wall architecture of *Candida* species and the high adaptive capacity of oral streptococci [18,19,20,21].

Overall, the data point to a preparation- and species-dependent antimicrobial effect, and i-PRF showed the most consistently broad inhibitory profile in this agar diffusion setting.

The antimicrobial activity observed in the present study is consistent with the established role of platelets as active components of innate immunity that release antimicrobial mediators and interact directly with pathogens [1,2,3,11,22,23].

Differences in antimicrobial activity between PRP, PRF, and i-PRF are likely related to their distinct cellular composition and fibrin architecture. Differences in preparation protocols have been shown to affect composition and biological activity, which may contribute to variation in antimicrobial outcomes across studies [24]. i-PRF is produced using low centrifugal forces and retains a liquid consistency due to its loosely organized fibrin network [25,26]. As a consequence of this preparation protocol, i-PRF typically contains higher concentrations of platelets and leukocytes compared with PRP and solid PRF preparations consistent with the low-speed concept described in the literature [27,28]. Both cell populations are known to contribute to antimicrobial activity through the release of bioactive mediators, including antimicrobial peptides, proteolytic enzymes, cytokines, and reactive oxygen species, which may collectively enhance antimicrobial activity [23].

In contrast, PRF forms a dense fibrin matrix that entraps platelets and leukocytes and facilitates a slower, sustained release of bioactive factors. While this property is beneficial for tissue regeneration and wound healing, it may restrict the immediate diffusion of antimicrobial substances in agar diffusion assays, thereby potentially leading to smaller initial inhibition zones [5]. This effect may be further reinforced by platelet-mediated clot retraction, which increases fibrin density and reduces pore size, thereby altering the mechanical and transport properties of fibrin-rich matrices [29]. PRP, although platelet-rich, generally contains fewer leukocytes and lacks a structured fibrin scaffold, which may further influence both the magnitude and duration of its antimicrobial effects [13].

The present findings are largely consistent with previous reports demonstrating antimicrobial activity of platelet-derived preparations. Recent comprehensive reviews, including antibiotic-loaded PRF systems and umbrella evaluations of PRP/PRF clinical applications, further support the broader relevance of platelet concentrates in antimicrobial research, although definitive clinical evidence is still lacking [30]. Drago et al. reported inhibitory effects of PRP against several bacterial and fungal species, albeit with marked interspecies variability [13]. Other investigations have likewise suggested that leukocyte- and platelet-rich concentrates can exhibit antimicrobial activity. For example, leukocyte- and platelet-rich plasma has been shown to inhibit growth of *S. aureus*, *E. faecalis*, and *Pseudomonas aeruginosa* in vitro in agar diffusion assays [31]. A recent scoping review noted antimicrobial effects of platelet-rich plasma (PRP) and PRF across multiple models and attributed these activities in part to the release of bioactive mediators, including antimicrobial proteins and reactive species [32]. Nevertheless, direct comparative analyses of PRP, PRF, and i-PRF—particularly with respect to oral pathogens—remain scarce, underscoring the relevance of the present study. It should be noted that the present study did not directly assess cellular composition or fibrin architecture of the preparations; therefore, these mechanistic considerations represent plausible hypotheses based on previous literature rather than confirmed mechanisms.

Variability between studies may be explained by differences in preparation protocols, centrifugal forces, cellular composition, and experimental methodology [25]. Diffusion-based assays, in particular, are strongly influenced by the physical properties of the tested materials, which may partially account for divergent results regarding the antimicrobial performance of fibrin-rich preparations [33].

### 3.3. Methodological Considerations and Clinical Relevance

Although the present study provides in vitro evidence for the antimicrobial potential of platelet concentrates, its findings cannot be directly extrapolated to clinical conditions. Factors such as saliva flow, host immune responses, tissue interactions, and the complex structure of polymicrobial biofilms are not adequately represented in agar diffusion models [34]. Moreover, inhibition zone size in agar diffusion assays is strongly influenced by the diffusion and release kinetics of the tested materials in the agar matrix and therefore does not directly reflect minimum inhibitory concentrations or antimicrobial efficacy against established biofilms [33]. The agar diffusion assay was selected as a standardized and widely used screening method for comparative evaluation across multiple microorganisms, but it does not provide quantitative information on bacterial killing or growth reduction in suspension. Furthermore, this approach does not allow quantitative determination of viable cell counts (e.g., CFU/mL) or microscopic assessment of cellular damage or biofilm structure. Future studies should therefore complement agar diffusion testing with quantitative broth dilution or CFU-based assays and microscopic analyses to obtain a more comprehensive characterization of antimicrobial activity. Importantly, agar diffusion assays inherently favor liquid formulations such as i-PRF, whereas fibrin-rich and solid preparations (e.g., PRF) may show reduced diffusion and consequently smaller inhibition zones independent of their true antimicrobial potential. In this context, fibrin-rich preparations such as PRF may be underestimated due to delayed and sustained release of bioactive factors from the fibrin matrix [5].

In addition, microorganisms were cultured under species-specific incubation conditions (aerobic, anaerobic, or CO_2_-enriched atmospheres) on appropriate solid media to ensure optimal (not too slow, not too fast) growth, but still with unavoidable variations in growth kinetics and substance diffusion behavior. Intrinsic differences in microbial growth rates are known to affect the extent and appearance of growth inhibition and may therefore contribute to variability in inhibition zone size [33]. Moreover, due to the biological nature and diffusion behaviour of platelet concentrates, antimicrobial activity does not always manifest as sharply demarcated clearance zones but may appear as clearly reduced growth, which represents a methodological characteristic rather than subjective interpretation [16]. Finally, the limited number of donors represents an additional limitation. Donor-related biological variability in platelet and leukocyte composition may have influenced the magnitude of inhibition zones; however, the study was not designed or powered to perform per-donor comparative analyses, and results were therefore analyzed across pooled donor samples. The limited sample size reflects the exploratory character of this study and should be considered when interpreting the findings.

Another limitation is the lack of quantitative characterization of platelet and leukocyte counts or growth factor concentrations in the individual preparations. All platelet concentrates were used within a short time window (≤30 min) after preparation to preserve biological activity, which precluded parallel compositional analyses without additional sample processing. Any such characterization would have required separate reserve aliquots and would therefore not necessarily reflect the material directly applied in the antimicrobial assays. Future studies should include defined reserve aliquots (e.g., cryopreserved at −80 °C) to enable parallel compositional characterization. The use of multiple statistical tests without a global correction for multiple comparisons represents an additional limitation. However, the study was designed as an exploratory investigation to identify general patterns and trends rather than to test predefined hypotheses.

Future studies should therefore include larger donor cohorts and employ alternative antimicrobial models, including biofilm-based systems or broth dilution assays, to more closely approximate clinical conditions and the complex architecture of oral microbial communities [35]. From a practical research perspective, future studies could implement a stepwise study design: (i) standardized preparation of PRP/PRF/i-PRF with parallel compositional characterization (platelet/leukocyte counts and selected growth factor markers), (ii) complementary antimicrobial testing using quantitative CFU-based or broth dilution assays and biofilm models, and (iii) verification in clinically relevant ex vivo or translational settings, followed by controlled clinical trials assessing efficacy and safety.

Overall, the present findings highlight the multifactorial nature of the antimicrobial activity of platelet concentrates. The observed differences between preparations and microorganisms underscore the importance of considering both biological composition and methodological context when interpreting in vitro antimicrobial data. Within these limitations, the results contribute to a better understanding of how platelet-derived products may support infection control in dental applications and provide a rationale for further translational and clinical investigations. The growing interest in alternative and biologically based antimicrobial approaches in dentistry, including natural products and adjunctive strategies for plaque control, further underscores the need to explore platelet-derived preparations as potential supportive tools [36]. From a clinical perspective, platelet concentrates could be explored as adjunctive measures in the management of periodontal and peri-implant infections, in regenerative procedures with increased infection risk, or as local antimicrobial support in endodontic and oral surgical interventions. In clinical practice, such preparations could be applied locally at the treatment site, for example, as injectable i-PRF or PRF membranes placed into periodontal or peri-implant defects, extraction sockets, or endodontic access cavities, where they may provide both regenerative support and local antimicrobial activity. However, it must be emphasized that the present findings are based solely on in vitro experiments. The clinical efficacy and safety of platelet concentrates for antimicrobial purposes remain untested and should be evaluated in well-designed, randomized controlled clinical trials.

## 4. Materials and Methods

### 4.1. Blood Collection

Venous blood was collected from five systemically healthy, non-smoking volunteers (2 female, 3 male; age range 25–45 years) who had not received antibiotics within the previous six months. Additional exclusion criteria were pregnancy and known coagulation disorders. Blood was drawn by antecubital venipuncture using a sterile butterfly cannula (Vacuette^®^ Evoprotect 21G × 19, Greiner Bio-One GmbH, Kremsmünster, Austria) into sterile tubes. Immediately after collection, the blood was processed in the laboratory as described in Section 4.2 to produce the corresponding platelet concentrates. All blood samples were collected at approximately the same time of day (around noon) to ensure standardized experimental conditions.

The protocol was approved by the local ethics committee (CTC-A-Nr.21-329, EK 379/21, RWTH Aachen University) and conducted in accordance with the Declaration of Helsinki (Fortaleza, 2013). All participants provided written informed consent, and samples were anonymized prior to analysis.

### 4.2. Preparation of Platelet Concentrates

PRP was prepared using commercially available plastic tubes containing sodium citrate as an anticoagulant and an integrated separation gel (Vi PRP-PRO tubes, Vi Medical GmbH, Bingen am Rhein, Germany). Whole blood was subjected to a single centrifugation step (single-spin protocol) using an Eppendorf 5810R centrifuge (Eppendorf AG, Hamburg, Germany) at 1200× *g* for 7 min, according to the recommended protocol. Following centrifugation, the PRP fraction located above the separation layer was carefully aspirated using sterile disposable syringes (Braun Injekt^®^, B. Braun, Melsungen, Germany), avoiding contamination with erythrocytes or leukocyte-rich fractions. PRP was used immediately for subsequent experiments.

PRF was prepared using sterile glass tubes designed for advanced PRF protocols (Choukroun’s A-PRF™ tubes, mectron Deutschland Vertriebs GmbH, Cologne, Germany). Whole blood was collected without anticoagulants, and coagulation was initiated immediately upon contact with the glass surface. Centrifugation was performed immediately after blood collection using a single-spin protocol at 700× *g* for 8 min following the manufacturer’s recommendations. After centrifugation, a fibrin clot was obtained in the upper portion of the tube. The clot was gently removed using sterile forceps, and residual red blood cells adhering to the lower part of the clot were carefully trimmed off to obtain a standardized PRF matrix.

i-PRF was prepared using dedicated glass tubes for i-PRF (Choukroun’s i-PRF™ tubes, mectron Deutschland Vertriebs GmbH). Whole blood was collected without anticoagulants and centrifuged immediately at low relative centrifugal force using the single-spin protocol at 400× *g* for 3 min, resulting in a liquid, platelet- and leukocyte-rich fraction in the upper layer of the tube. This fraction was carefully aspirated using sterile disposable syringes and processed immediately to preserve its injectable consistency.

All platelet concentrates were prepared by the same operator using the same centrifuge device to minimize inter-operator and inter-device variability. No quantitative characterization of platelet and leukocyte counts or growth factor concentrations was performed for the individual preparations in this cohort. All preparations were used within 30 min after processing.

### 4.3. Bacterial Strains

A panel of clinically relevant oral microorganisms was selected for the antimicrobial assays, including *A. actinomycetemcomitans*, *P. gingivalis*, *P. intermedia*, *S. aureus*, *S. mutans*, *S. mitis*, *E. faecalis* and *C. albicans*. The selected microorganisms represent well-established pathogens or opportunistic species commonly associated with oral and periodontal infections. *S. aureus* was included because it is frequently detected in oral and peri-implant infections and represents an opportunistic pathogen of increasing relevance in dentistry. Clinical isolates of *C. albicans* and *E. faecalis* were used to better reflect clinically encountered strains and their potential variability compared with long-term laboratory reference strains, and because both species are frequently implicated in persistent and recurrent endodontic infections. The corresponding ATCC reference numbers are provided in Table 2. For microorganisms available only as clinical isolates from the University Hospital RWTH Aachen, internal strain identifiers (OMI numbers) are listed instead. Reference strains or clinical isolates were cultivated under species-specific conditions as summarized in Table 2. Mueller–Hinton agar (Thermo Scientific, Waltham, MA, USA PB5007A) was used for culturing *C. albicans* because it is a standardized medium recommended for agar diffusion–based antimicrobial susceptibility testing of yeasts and provides reproducible growth and reliable diffusion characteristics. Individual colonies from fresh agar plates were suspended in sterile saline or appropriate culture medium and adjusted to a turbidity equivalent to 0.5 McFarland (~1–2 × 10^8^ CFU/mL). Prior to preparation of the inoculum, purity of the cultures was verified by routine light microscopic examination and Gram staining to confirm the absence of contamination and the expected microbial morphology. After even distribution of the standardized inoculum (200 µL with approximately 10^7^ CFU) using a sterile Drigalski spatula (Carl Roth GmbH+Co.KG, Karlsruhe, Germany) (to later create a lawn of semi- confluent colonies), the plates were pre-incubated for 2–4 h, depending on the microorganism (Table 2), to ensure metabolic activity and timely entry into the logarithmic growth phase prior to application of the test substances, taking into account species-specific differences in growth kinetics and generation times to achieve a homogeneous but not completely confluent growth layer. The selected pre-incubation times represent the shortest incubation period that reproducibly resulted in visible, homogeneous growth without full confluent overgrowth for each species, based on preliminary growth observations and routine laboratory experience.

### 4.4. Culture Media and Agar Plates

Different agar media were used according to the specific requirements of each microorganism; the respective assignments of media to strains are summarized in Table 2. Tryptic Soy Agar with 5% sheep blood (TSASB) plates (Thermo Scientific, PB5012A) and Mueller–Hinton blood agar plates were used. Mueller–Hinton II Broth (Becton, Dickinson and Company (Franklin Lakes, NJ, USA), BD 212322), Bacto Agar (Becton, Dickinson and Company (Franklin Lakes, NJ, USA), BD 214010), Brucella Broth (Becton, Dickinson and Company (Franklin Lakes, NJ, USA), BD 211088) and Vitamin K1–Hemin solution (Becton, Dickinson and Company (Franklin Lakes, NJ, USA), BD 212354) were applied as required. For in-house prepared agar plates, Mueller–Hinton agar was prepared using 11 g MH base (Becton, Dickinson and Company (Franklin Lakes, NJ, USA), BD 212322) and 8 g agar in 500 mL distilled water, while Brucella agar plates were prepared using 14 g Brucella base (Becton, Dickinson and Company (Franklin Lakes, NJ, USA)) and 9 g agar in 500 mL distilled water supplemented with 5 mL Vitamin K1–Hemin solution.

### 4.5. Agar Diffusion Test

The agar diffusion assay was performed based on the standardized EUCAST disk diffusion methodology and quality control recommendations for antimicrobial susceptibility testing [37] but with modifications necessary. After completion of the pre-incubation period, 5 µL of each liquid test substance (PRP and i-PRF), as well as 5 µL of the control substances NaCl (0.9% sodium chloride solution, B. Braun SE, Melsungen, Germany) and chlorhexidine (CHX, 2%, clinical stock solution, University Hospital RWTH Aachen, Aachen, Germany), were applied to the agar surface. Amoxicillin (10 µg per disc, Oxoid Ltd., Basingstoke, UK) was used as an additional positive control for bacterial strains. For *C. albicans*, only chlorhexidine served as positive control, as amoxicillin is not active against yeasts. For PRF, the fibrin clot was gently compressed between sterile gauze to remove excess serum, cut into small standardized fragments (approximately 3–5 mm in diameter), and individual fragments were transferred using either a sterile pipette tip with an enlarged opening or sterile forceps and placed directly onto the agar surface. The plates were subsequently incubated for an additional 24 to 72 h, depending on the microorganism, under species-specific culture conditions (Table 2).

Digital images of the agar plates were acquired at the final incubation time point for each microorganism. Inhibition zone diameters measured at this time point were used for statistical analysis. Zones of antimicrobial activity were defined as areas of complete growth inhibition or zones showing markedly reduced growth compared with the surrounding lawn. Measurements were performed in diameter of inhibition zone (in mm) using digital image analysis software (FIJI—Fiji Is Just ImageJ, version 2.16.0/1.54p, ImageJ2 framework; ImageJ.net/Fiji; based on ImageJ, National Institutes of Health, Bethesda, MD, USA) by an examiner blinded to the experimental groups. For each plate, the diameter was measured in two perpendicular directions (top–bottom and left–right), and the mean value was calculated. Each measurement was performed in triplicate, and the resulting mean growth inhibition zone diameter (in mm) was used for statistical analysis as described in Section 4.6.

To assess measurement reliability, approximately 11% of the inhibition zones from a representative subset of plates covering different microorganisms, test preparations and inhibition magnitudes were independently re-measured by a second blinded examiner. Inter-observer agreement was excellent, with a mean absolute difference of 0.89 ± 0.74 mm. Measurements of plates without detectable inhibition zones were identical between observers. The measurements of both examiners showed a very strong correlation (r = 0.99, *p* < 0.001), confirming the robustness and reproducibility of the measurement procedure.

### 4.6. Statistical Analysis

Inhibition zone diameters (mm) were averaged from technical triplicates per donor, preparation, and microorganism, and these donor-level mean values were used for statistical analysis. As the data were non-normally distributed, non-parametric statistical tests were used. Differences between preparations were assessed using Kruskal–Wallis tests with post hoc Mann–Whitney U tests where appropriate. For Mann–Whitney U post hoc comparisons, effect sizes were calculated using r = Z/√N. Differences between microorganisms were evaluated using Friedman tests. Results are reported as medians and interquartile ranges (IQR). Statistical analyses were performed using IBM SPSS Statistics 30 (BM Corp., Armonk, NY, USA), with *p* < 0.05 considered statistically significant. The sample size was determined based on the exploratory nature of this in vitro study and on previous comparable investigations evaluating the antimicrobial effects of platelet concentrates, which typically included between three and ten donors. A formal a priori power calculation was not performed, as the primary aim was to identify general susceptibility patterns and preparation-dependent trends rather than to detect small effect sizes. Given the exploratory nature of this study and the limited sample size, no global adjustment for multiple comparisons was applied. Bonferroni correction was used only for selected post hoc pairwise comparisons. All statistical analyses should therefore be considered exploratory and interpreted accordingly.

## 5. Conclusions

Within the limitations of this in vitro study, platelet concentrates demonstrated measurable antimicrobial activity against clinically relevant oral pathobionts in agar diffusion assays. This activity was not uniform but depended on both the type of platelet preparation and the microbial species tested. Among the investigated preparations, i-PRF exhibited the most consistent and pronounced antimicrobial effects, whereas PRP and PRF showed smaller and more variable inhibitory profiles.

These findings suggest that the antimicrobial potential of platelet concentrates is closely linked to their cellular composition and release characteristics, rather than representing a nonspecific effect. Although the results cannot be directly extrapolated to clinical conditions, they support the concept that platelet-derived products—particularly i-PRF—may offer adjunctive antimicrobial benefits in dental therapy. Further studies employing larger donor cohorts, biofilm-based models, and clinical investigations are warranted to clarify the translational relevance of these effects.

## Figures and Tables

**Table 1:** Kruskal–Wallis *p*-values for comparisons between preparations sorted by microorganism statistically significant values in bold). Post hoc pairwise comparisons were conducted only for *E. faecalis*. * Post hoc testing was not applicable for *C. albicans* because no variability was observed in the PRP and PRF groups (all values = 0 mm).

Microorganism	*p*-Values	Post Hoc	
*A. actinomycetemcomitans*	0.826	–	
*S. mutans*	0.766	–	
*S. mitis*	0.688	–	
*P. gingivalis*	0.574	–	
*P. intermedia*	0.566	–	
*S. aureus*	0.067	–	
*C. albicans*	**0.007**	*	
*E. faecalis*	**0.007**	PRP vs. PRF	0.31
		PRP vs. i-PRF	**0.008**
		PRF vs. i-PRF	**0.008**

**Table 2:** Investigated microorganisms with corresponding ATCC or internal OMI strain identifiers and individual culture conditions. After inoculation and before positioning the platelet concentrates, agar plates were pre-incubated at 37 °C under optimal atmospheric conditions to ensure activation of bacterial cells (end of lag-phase).

Microorganism	Strain	Medium	Atmosphere	Pre-Incubation	Incubation
*P. gingivalis*	ATCC 33277 T	Brucella agar	anaerobic	4 h	72 h
*P. intermedia*	ATCC 25611	Brucella agar	anaerobic	4 h	72 h
*A. actinomycetemcomitans*	ATCC 33384 T	Mueller–Hinton agar with blood	candle jar	3 h	72 h
*S. mitis*	ATCC 49456 T	Mueller–Hinton agar with blood	5% CO_2_	3 h	48 h
*S. mutans*	ATCC 25175	Mueller–Hinton agar with blood	5% CO_2_	3 h	48 h
*C. albicans*	OMI 286	Mueller–Hinton agar	aerobic	2 h	24 h
*E. faecalis*	OMI 563	Mueller–Hinton agar	aerobic	2 h	24 h
*S. aureus*	ATCC 25923	Mueller–Hinton agar	aerobic	2 h	24 h

## Data Availability

The datasets used and/or analyzed during the current study are available from the corresponding author on reasonable request.

## Supplementary Materials

The following supporting information can be downloaded at: https://www.mdpi.com/article/10.3390/antibiotics15020173/s1, Supplementary Figure S1: Representative inhibition patterns for all tested microorganisms.

## Abbreviations

The following abbreviations are used in this manuscript:

*A. actinomycetemcomitans*	*Aggregatibacter actinomycetemcomitans*
*C. albicans*	*Candida albicans*
CFU	colony-forming units
CHX	chlorhexidine
DNA	deoxyribonucleic acid
*E. faecalis*	*Enterococcus faecalis*
i-PRF	injectable platelet-rich fibrin
IQR	interquartile range
L-PRF	leukocyte- and platelet-rich fibrin
NaCl	sodium chloride
NETs	neutrophil extracellular traps
*P. gingivalis*	*Porphyromonas gingivalis*
*P. intermedia*	*Prevotella intermedia*
PRF	platelet-rich fibrin
PRP	platelet-rich plasma
ROS	reactive oxygen species
*S. aureus*	*Staphylococcus aureus*
*S. mitis*	*Streptococcus mitis*
*S. mutans*	*Streptococcus mutans*
TLR	toll-like receptor

## References

[1] Groeger S.E., Meyle J. (2015). Epithelial Barrier and Oral Bacterial Infection. Periodontol. 2000.

[2] Lalla E., Papapanou P.N. (2011). Diabetes Mellitus and Periodontitis: A Tale of Two Common Interrelated Diseases. Nat. Rev. Endocrinol..

[3] World Health Organization (2014). Antimicrobial Resistance: Global Report on Surveillance.

[4] Marx R.E. (2001). Platelet-Rich Plasma (PRP): What Is PRP and What Is Not PRP?. Implant. Dent..

[5] Dohan Ehrenfest D.M., Rasmusson L., Albrektsson T. (2009). Classification of Platelet Concentrates: From Pure Platelet-Rich Plasma (P-PRP) to Leucocyte- and Platelet-Rich Fibrin (L-PRF). Trends Biotechnol..

[6] Miron R.J., Zucchelli G., Pikos M.A., Salama M., Lee S., Guillemette V., Fujioka-Kobayashi M., Bishara M., Zhang Y., Wang H.-L. (2017). Use of Platelet-Rich Fibrin in Regenerative Dentistry: A Systematic Review. Clin. Oral Investig..

[7] Choukroun J., Diss A., Simonpieri A., Girard M.-O., Schoeffler C., Dohan S.L., Dohan A.J.J., Mouhyi J., Dohan D.M. (2006). Platelet-Rich Fibrin (PRF): A Second-Generation Platelet Concentrate. Part V: Histologic Evaluations of PRF Effects on Bone Allograft Maturation in Sinus Lift. Oral Surg. Oral Med. Oral Pathol. Oral Radiol. Endod..

[8] Jansen E.E., Braun A., Jansen P., Hartmann M. (2021). Platelet-Therapeutics to Improve Tissue Regeneration and Wound Healing—Physiological Background and Methods of Preparation. Biomedicines.

[9] Semple J.W., Italiano J.E., Freedman J. (2011). Platelets and the Immune Continuum. Nat. Rev. Immunol..

[10] Koupenova M., Clancy L., Corkrey H.A., Freedman J.E. (2018). Circulating Platelets as Mediators of Immunity, Inflammation, and Thrombosis. Circ. Res..

[11] Yeaman M.R. (2014). Platelets: At the Nexus of Antimicrobial Defence. Nat. Rev. Microbiol..

[12] Aggour R.L., Gamil L. (2017). Antimicrobial Effects of Platelet-Rich Plasma against Selected Oral and Periodontal Pathogens. Pol. J. Microbiol..

[13] Drago L., Bortolin M., Vassena C., Taschieri S., Del Fabbro M. (2013). Antimicrobial Activity of Pure Platelet-Rich Plasma against Microorganisms Isolated from Oral Cavity. BMC Microbiol..

[14] Yang L.-C., Hu S.-W., Yan M., Yang J.-J., Tsou S.-H., Lin Y.-Y. (2015). Antimicrobial Activity of Platelet-Rich Plasma and Other Plasma Preparations against Periodontal Pathogens. J. Periodontol..

[15] Badade P.S., Mahale S.A., Panjwani A.A., Vaidya P.D., Warang A.D. (2016). Antimicrobial Effect of Platelet-Rich Plasma and Platelet-Rich Fibrin. Indian J. Dent. Res..

[16] Moraschini V., Miron R.J., Mourão C.F.D.A.B., Louro R.S., Sculean A., da Fonseca L.A.M., Calasans Maia M.D., Shibli J.A. (2024). Antimicrobial Effect of Platelet-rich Fibrin: A Systematic Review of in Vitro Evidence-based Studies. Periodontol. 2000.

[17] Jasmine S., Thangavelu A., Janarthanan K., Krishnamoorthy R., Alshatwi A.A. (2020). Antimicrobial and Antibiofilm Potential of Injectable Platelet Rich Fibrin—A Second-Generation Platelet Concentrate—Against Biofilm Producing Oral Staphylococcus Isolates. Saudi J. Biol. Sci..

[18] Brown G.D., Denning D.W., Gow N.A.R., Levitz S.M., Netea M.G., White T.C. (2012). Hidden Killers: Human Fungal Infections. Sci. Transl. Med..

[19] Peschel A., Sahl H.-G. (2006). The Co-Evolution of Host Cationic Antimicrobial Peptides and Microbial Resistance. Nat. Rev. Microbiol..

[20] Maisetta G., Brancatisano F.L., Esin S., Campa M., Batoni G. (2011). Gingipains Produced by Porphyromonas Gingivalis ATCC49417 Degrade Human-β-Defensin 3 and Affect Peptide’s Antibacterial Activity in Vitro. Peptides.

[21] Kreth J., Merritt J., Qi F. (2009). Bacterial and Host Interactions of Oral Streptococci. DNA Cell Biol..

[22] Tang Y.Q., Yeaman M.R., Selsted M.E. (2002). Antimicrobial peptides from human platelets. Infect Immun..

[23] Kraemer B.F., Campbell R.A., Schwertz H., Cody M.J., Franks Z., Tolley N.D., Kahr W.H.A., Lindemann S., Seizer P., Yost C.C. (2011). Platelets in Infectious Disease. Nat. Med..

[24] Uyeda F.H., Vargas G.Q., Malavazi L.M., Macedo T.T., Gomes A.P.D.A.P., Bueno M.R., Tolentino P.H.M.P., Silva L.D.A.D., Figueiredo L.C., Shibli J.A. (2025). Platelet-Rich Fibrin Obtained from Different Protocols Affects the Formation of the in Vitro Multispecies Subgingival Biofilm Associated with Periodontitis. J. Oral Microbiol..

[25] Ghanaati S., Booms P., Orlowska A., Kubesch A., Lorenz J., Rutkowski J., Landes C., Sader R., Kirkpatrick C., Choukroun J. (2014). Advanced Platelet-Rich Fibrin: A New Concept for Cell-Based Tissue Engineering by Means of Inflammatory Cells. J. Oral Implantol..

[26] Miron R.J., Fujioka-Kobayashi M., Bishara M., Zhang Y., Hernandez M., Choukroun J. (2017). Injectable Platelet Rich Fibrin (i-PRF): Opportunities in Regenerative Dentistry?. Clin. Oral Investig..

[27] Choukroun J., Ghanaati S. (2018). Reduction of Relative Centrifugation Force within Injectable Platelet-Rich-Fibrin (PRF) Concentrates Advances Patients’ Own Inflammatory Cells, Platelets and Growth Factors: The First Introduction to the Low Speed Centrifugation Concept. Eur. J. Trauma Emerg. Surg..

[28] Farshidfar N., Amiri M.A., Estrin N.E., Ahmad P., Sculean A., Zhang Y., Miron R.J. (2025). Platelet-rich Plasma (PRP) versus Injectable Platelet-rich Fibrin (I-PRF): A Systematic Review across All Fields of Medicine. Periodontol. 2000.

[29] Jansen E.E., Hartmann M. (2021). Clot Retraction: Cellular Mechanisms and Inhibitors, Measuring Methods, and Clinical Implications. Biomedicines.

[30] Niemczyk W., Żurek J., Niemczyk S., Kępa M., Zięba N., Misiołek M., Wiench R. (2025). Antibiotic-Loaded Platelet-Rich Fibrin (AL-PRF) as a New Carrier for Antimicrobials: A Systematic Review of In Vitro Studies. Int. J. Mol. Sci..

[31] Cieślik-Bielecka A., Bold T., Ziółkowski G., Pierchała M., Królikowska A., Reichert P. (2018). Antibacterial Activity of Leukocyte- and Platelet-Rich Plasma: An In Vitro Study. BioMed Res. Int..

[32] Karan C., Jeyaraman M., Jeyaraman N., Ramasubramanian S., Khanna M., Yadav S. (2023). Antimicrobial Effects of Platelet-Rich Plasma and Platelet-Rich Fibrin: A Scoping Review. Cureus.

[33] Balouiri M., Sadiki M., Ibnsouda S.K. (2016). Methods for in Vitro Evaluating Antimicrobial Activity: A Review. J. Pharm. Anal..

[34] Stewart P.S., William Costerton J. (2001). Antibiotic Resistance of Bacteria in Biofilms. Lancet.

[35] Donlan R.M., Costerton J.W. (2002). Biofilms: Survival Mechanisms of Clinically Relevant Microorganisms. Clin. Microbiol. Rev..

[36] Lile I.E., Hajaj T., Veja I., Hosszu T., Vaida L.L., Todor L., Stana O., Popovici R.-A., Marian D. (2025). Comparative Evaluation of Natural Mouthrinses and Chlorhexidine in Dental Plaque Management: A Pilot Randomized Clinical Trial. Healthcare.

[37] EUCAST (2025). The EUCAST Disk Diffusion Method for Antimicrobial Susceptibility Testing.

