# Supplementary material for: In Vitro Antimicrobial Potential of Different Platelet Concentrates Against Eight Clinically Relevant Oral Pathobionts
Source: Antibiotics (Basel). 2026 Feb 5;15(2):173. doi: 10.3390/antibiotics15020173 (PMC12937402; doi:10.3390/antibiotics15020173)

Supplementary Figure S1. Representative inhibition patterns for all tested microorganisms.

Representative agar plate images showing antimicrobial effects of the control substances (chlorhexidine (CHX) as positive control and 0.9% NaCl as negative control), as well as injectable platelet-rich fibrin (i-PRF), platelet-rich fibrin (PRF), and platelet-rich plasma (PRP) against the tested microorganisms, displayed in a consistent left-to-right order. Images illustrate typical inhibition patterns and are provided for visualization only; quantitative analysis and statistics were based on digitally measured inhibition zone diameters as described in the Methods section. Inhibition zones were measured as diameters in two perpendicular directions and averaged, with mean values from technical triplicates used for analysis. Images are presented in grayscale to improve contrast; brightness and contrast were uniformly adjusted without altering structural image information. The images show whole agar plates from representative experiments and do not necessarily depict plates with the largest inhibition zones, but rather typical examples of observed patterns.

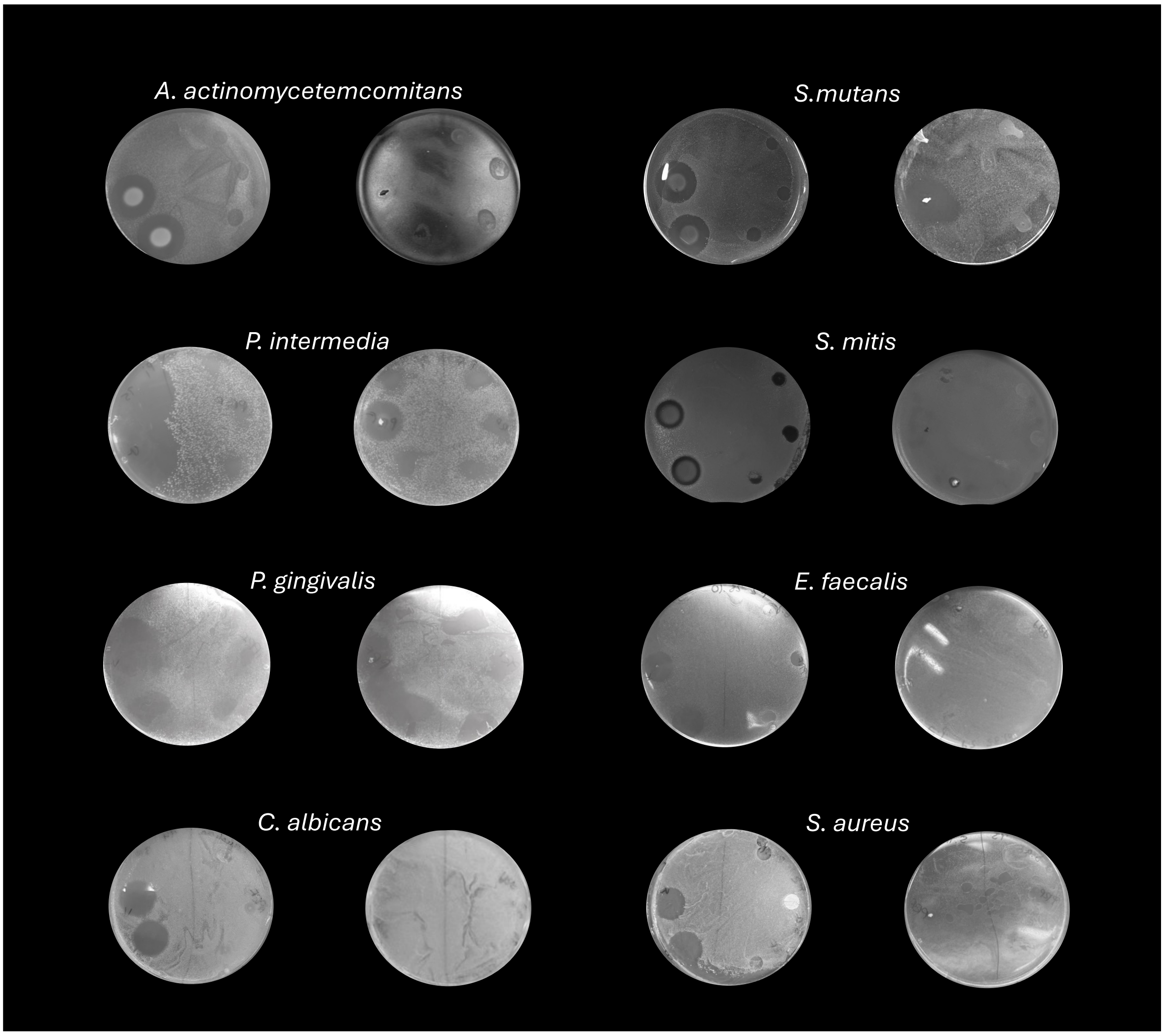

Supplement: Supplementary file 1 [file antibiotics-15-00173-s001.zip › antibiotics-4120535-supplementary.pdf]
